# Association Between Gastroesophageal Reflux Disease Severity and Depression and Anxiety in Young Adults: A Cross-Sectional Study

**DOI:** 10.7759/cureus.92993

**Published:** 2025-09-23

**Authors:** Maheen Asim, Hareema Saeed Khan, Muhammad Hammad Khan, Rabbia Aslam, Anam Fatima, Nitasha Khan

**Affiliations:** 1 Internal Medicine, Federal Government Poly Clinic Hospital, Islamabad, PAK; 2 Medicine, Mayo Hospital, Lahore, PAK

**Keywords:** anxiety, cross-sectional study, depression, gastroesophageal reflux disease, young population

## Abstract

Background and objective

Healthcare professionals worldwide often encounter gastroesophageal reflux disease (GERD). With a rising incidence of anxiety and depression in young adults, it is essential to investigate these psychological aspects as potential risk factors for GERD and its exacerbation. Early screening and treatment of anxiety and depression can aid physicians in achieving better symptomatic control and improving overall quality of life. Hence, this study was carried out to determine the frequency of anxiety and depression in young adults with symptoms of GERD. A cross-sectional study was conducted enrolling patients from the Federal Government Polyclinic Hospital, Islamabad, between January 1, 2023, and June 30, 2023.

Methods

Patients with gastrointestinal symptoms were enrolled. GERD diagnosis was made using the frequency scale for the symptoms of GERD (FSSG) questionnaire, and anxiety or depression was assessed using General Anxiety Disorder-7 (GAD-7) and Patient Health Questionnaire-9 (PHQ-9) scales. Possible confounding factors, such as age, gender, BMI, smoking, education, and socioeconomic status, were controlled for through stratification. In our primary analysis, a chi-square test was applied to assess the association of anxiety and depression with GERD. The data were analyzed using IBM SPSS Statistics version 21.0 (Armonk, NY: IBM Corp.) for Windows.

Results

We obtained data from 210 patients with a mean age of 28.8±6.6 years. Out of these, 122 (58.1%) were female and 88 (41.9%) were male. GERD was found among 133 (63.3%) patients. Furthermore, in this study, the frequency of anxiety and depression was found to be 39 (29.3%) and 99 (74.4%), respectively, out of the individuals diagnosed with GERD. Anxiety and depression were significantly associated with the development of GERD in our population.

Conclusion

Young adults with GERD should be screened for anxiety and depression, as there is a significant association between these psychological conditions and GERD.

## Introduction

Gastroesophageal reflux disease (GERD) is a chronic digestive disorder caused by the reflux of acidic stomach contents into the esophagus. It is frequently encountered by healthcare professionals globally. The prevalence of GERD has risen in Eastern Asia, suggesting a concerning trend. Southeast Asia and the Middle East face a heavier burden of GERD, emphasizing the need for region-specific healthcare strategies [1,2]. In the Western world, the prevalence is even greater, with approximately 30% of the population affected by GERD, compared to less than 10% in Asia [3].

GERD can often lead to either bothersome symptoms or potential complications [4]. Typical symptoms like heartburn and regurgitation are the hallmark of GERD, but atypical symptoms, such as dysphagia, chest pain, water brash (hypersalivation), chronic cough, and hoarseness can occur as well [5]. Such symptoms are irritating and can harm the physical, mental, and social well-being of the patient, significantly affecting their ability to cope with daily life activities [6,7]. The psychoemotional impact of GERD has been demonstrated using reliable quality-of-life instruments and has shown a higher susceptibility of such patients to psychological disorders, including depression [8]. Psychological issues like emotional stress can affect the functioning of the gastrointestinal tract as well, leading to gastrointestinal symptoms and disease. Several studies have demonstrated the influence of psychological factors on the severity of gastrointestinal disorders by altering pain perception via the gut-brain axis, a mechanism that may also contribute to GERD [9,10]. Overstimulation of the gut-brain axis occurs in states of anxiety and stress, which can lead to worsening GERD symptoms and further worry about gut health, leading to a self-perpetuating cycle [11].

The impact of psychological stressors on GERD has been studied in the literature. A cross-sectional study, enrolling 2,500 participants from the younger population ranging from ages 18 to 40 years in Karachi, indicated a notably higher occurrence of anxiety and depression in individuals with GERD when compared to those without GERD, emphasizing the higher prevalence in this age group [12]. A similar cross-sectional study conducted in India demonstrated significantly greater prevalence of anxiety and depression among individuals with GERD among the young and old populations [13]. There is still uncertainty about the extent to which GERD is affected by anxiety and depression; a cohort study conducted in the Australian male population identified a robust link between GERD and anxiety but found no such association with depression [14].

The conflicting outcomes from these studies underscore the necessity for additional research to gain a more comprehensive knowledge of the relationship between GERD and psychological factors. This is even more important now, considering the increasing prevalence of anxiety and depression among young adults, which may induce or worsen GERD. Hence, the objective of our study is to examine the association of GERD with anxiety and depression and to understand other factors that may contribute to the greater prevalence of GERD in the adult population. Understanding this association and increasing clinical assessment of psychological stressors to mitigate them may serve to improve patient outcomes for those affected by GERD.

## Materials and methods

This cross-sectional study was conducted in the Department of Medicine, Federal Government Poly Clinic Hospital, Islamabad. Patients were enrolled using a non-probability consecutive sampling technique over a six-month period from January 1, 2023, to June 30, 2023. The target sample size was calculated as 210 using Raosoft sample size calculator (Seattle, WA: Raosoft Inc.) by keeping a confidence interval (CI) of 95%, an alpha value of 0.05, and 16% anticipated prevalence of GERD in the young population [12].

The inclusion criteria were children and adult patients of either gender, aged 13 to 40 years. Patients presenting to the Outpatient Department of Poly Clinic Hospital, Islamabad, with gastrointestinal complaints were evaluated for GERD using the frequency scale for the symptoms of GERD (FSSG) questionnaire as follows: patients with scores greater than eight on the scale were classified as having GERD [15]. Anxiety was defined as a score of 10 or greater on the GAD-7 scale [16]. Depression was evaluated using the PHQ-9 scale as follows: a score of five or greater was indicative of the patient suffering from depression [17].

The exclusion criteria were as follows: (1) patients with esophageal or gastric malignancy, previous gastrointestinal surgery, and peptic ulcer disease, (2) patients with chronic comorbidities, such as diabetes mellitus, ischemic heart disease, hypertension, asthma, and chronic obstructive pulmonary disease (COPD), (3) patients with diagnosed psychiatric illnesses or taking any psychiatric medications.

Data collection procedure

After getting approval from the hospital's ethical and research committee, patients presenting to the Outpatient Department of Federal Government Poly Clinic Hospital, Islamabad, with gastrointestinal symptoms were enrolled in the study. Written informed consent was obtained from each participant. Patients of both genders, aged 13-40 years, presenting with gastrointestinal complaints, were included. Demographic details of the patients were noted in a self-structured questionnaire. The outcome of the patients was noted in terms of the frequency of GERD in the population and the frequency of anxiety and depression among those patients. The patients were interviewed, and the physician performing the interview was responsible for filling out the questionnaires. All the data collection and study procedures were conducted by the researcher himself to maintain data continuity and quality. Details of questionnaires used in this study are provided in appendix 1.

Data analysis

Data were entered and analyzed using IBM SPSS Statistics version 21.0 (Armonk, NY: IBM Corp.) for Windows. The categorical data, including gender, education, socioeconomic status, and smoking status, were presented as descriptive statistics (frequencies and percentages). Similarly, patients were categorized based on the presence or absence of GERD, anxiety, and depression using the cutoffs mentioned above; the results were presented as frequencies and percentages. The quantitative variables, such as age and BMI, were presented as means and standard deviations. Possible confounding factors, such as age, gender, BMI, smoking, education, and socioeconomic status, were controlled for through stratification. In our primary analysis, a chi-square test was applied to assess the association of anxiety and depression with GERD. Univariate logistic regression was performed to evaluate the effect estimates of said association, reported as odds ratio (OR) and 95% confidence interval (CI). A correlation analysis was performed ad hoc to assess the relation between FSSG scores and anxiety/depression severity assessed using the GAD-7 and PHQ-9 scores. A p-value of less than 0.05 was considered significant for these analyses.

As a secondary analysis of the factors contributing to depression and anxiety, we evaluated whether patients belonging to certain demographic and socioeconomic groups had a greater incidence of these psychological illnesses compared to others. A chi-square test was again used to assess the association of gender, BMI, smoking, education, and socioeconomic status on patients having anxiety and depression. To assess the possibility of these factors confounding the association between anxiety/depression and GERD, a multivariate logistic regression was performed.

## Results

We obtained data from 210 patients with a mean age of 28.8±6.6 years. Of these, 122 (58.1%) were female and 88 (41.9%) were male. The FSSG score was used to diagnose GERD among patients. GERD was found among 133 (63.3%) patients (Table 1). A major proportion (93.3%) of the included patients were of normal weight (BMI between 18.5 and 24.9), and there was a significant representation of patients from the middle socioeconomic class (78.6%), defined as people earning between Rs. 60,000 and Rs. 200,000 at the time the study was conducted. Notably, a considerable proportion of patients (47.6%) had attained the highest level of educational attainment, which was matriculation or intermediate studies.

**Table 1:** Baseline characteristics of enrolled participants.

Characteristics		Frequency (%)
Gender	Male/female	122/88 (58.1/41.9)
Smoking	Yes/no	31/179 (14.8/85.2)
BMI	Underweight	14 (6.7)
	Normal	196 (93.3)
	Overweight	0 (0)
Socioeconomic status	Lower class	45 (21.4)
	Middle class	165 (78.6)
	Upper class	0 (0)
Education	Up to primary	26 (12.4)
	Matric or intermediate	100 (47.6)
	Graduate or post-graduate	84 (40.0)

Table 2 summarizes the distribution of GERD among patients by gender, smoking status, BMI, socioeconomic status, and educational level. To determine whether these factors influenced the development of GERD and potentially confounded the results, chi-square tests were conducted, which revealed no significant association between these factors and the development of GERD. Details of these analyses are provided in the appendix (appendix 2).

**Table 2:** Prevalence of GERD stratified for gender, smoking status, BMI, socioeconomic status, and education. GERD: gastroesophageal reflux disease

Characteristics		Frequency yes/no (percentage)
Gender	Female	83/39 (68.0/32.0)
	Male	50/38 (56.8/43.2)
Smoking	No	113/66 (63.1/36.9)
	Yes	20/11 (64.5/35.5)
BMI	Underweight	7/7 (50.0/50.0)
	Normal	126/70 (64.3/35.7)
Socioeconomic status	Lower class	30/15 (66.7/33.3)
	Middle class	103/62 (62.4/37.6)
	Upper class	0/0 (0.0/0.0)
Education	Up to primary	21/5 (80.8/19.2)
	Matric or intermediate	61/39 (61.0/39.0)
	Graduate or post-graduate	51/33 (60.7/39.3)

Primary analysis

Furthermore, in this study, the frequency of anxiety among GERD patients was found to be 39 (29.3%) as determined by the GAD-7 questionnaire. In order to determine the association of anxiety with GERD, the chi-square test of significance was performed, which yielded a significant association between the presence of anxiety in patients with GERD (Table 3). The strength of the association, measured using logistic regression, showed a very strong association between anxiety and GERD. However, the value of the effect estimate is likely overestimated due to the small sample size, and all of the patients with anxiety had GERD (100% of patients with anxiety had GERD) (appendix 3).

**Table 3:** Association between anxiety and development of GERD. Chi-square test of significance (n=210) = 27.73, p<0.01. GERD: gastroesophageal reflux disease; GAD-7: General Anxiety Disorder-7

Variables		GERD diagnosis	
		No	Yes
Anxiety according to GAD-7 scale	No	77	94
	Yes	0	39

The frequency of depression, as determined by the PHQ-9 questionnaire, was 99 out of 133 individuals who had GERD (74.4%). Depression was significantly and very strongly associated with the presence of GERD in these patients (OR: 212.80, 95% CI: 28.5-1588.5, p<0.001), as shown in Table 4 and the appendix (appendix 4).

**Table 4:** Association between depression and development of GERD. Chi-square test of significance (n=210) = 102.5, p<0.001. GERD: gastroesophageal reflux disease; PHQ-9: Patient Health Questionnaire-9

Variables		GERD diagnosis	
		No	Yes
Depression according to PHQ-9	No	76	35
	Yes	1	98

A significant positive correlation was observed between FSSG scores and anxiety severity measured using the GAD-7 questionnaire (Pearson’s r=0.418, Spearman correlation coefficient=0.492, p<0.001). There was also a significant positive correlation between FSSG questionnaire scores and PHQ-9 scores (Pearson’s r=0.415, Spearman correlation coefficient=0.442, p<0.001), indicating that GERD severity was linked with severity of anxiety and depressive symptoms (appendix 5).

Secondary analysis

When patients in various subcategories were compared on the basis of whether they developed anxiety, it was observed that there was a significant association between the level of educational attainment and anxiety. Those with a higher educational attainment level (matric/intermediate or graduate/post-graduate) were less likely to experience anxiety than those who had received education up to the primary level (appendix 6). No significant association was observed between baseline characteristics and depression among the patients.

Multivariate logistic regression for anxiety and its relation to GERD revealed BMI and gender to be significant factors affecting this association. However, none of these factors were identified as confounders for the association between depression and GERD (appendix 7).

## Discussion

This study shows that, with a diagnostic threshold of 10 on the GAD-7 questionnaire for anxiety, there is a significant association between anxiety and GERD. Furthermore, with a diagnostic threshold of 5 on the PHQ-9 questionnaire for depression, GERD is significantly associated with depression.

The prevalence of GERD in young adults has become a subject of increasing concern. A study conducted in tertiary clinics of Pakistan showed that 64% of patients with GERD are younger than 40 years [18]. This was significantly more than the study by Bai et al., which found GERD in up to 16% of the young adult population [12]. A growing body of research has explored its association with mental health conditions, such as depression and anxiety. However, the full understanding of the extent of this relationship remains limited. Firstly, lifestyle factors play a pivotal role in the increased prevalence of GERD among young adults. Changes in dietary habits, including the consumption of acidic and spicy foods, irregular eating patterns, and an increase in sedentary behaviors, contribute to the susceptibility to GERD. Additionally, the prevalence of obesity, a known risk factor for GERD, has been on the rise in younger age groups, further exacerbating the issue [19].

Zamani et al. conducted a meta-analysis and found that as many as one-third of individuals with GERD may encounter anxiety and depression. Understanding the reciprocal relationship between these psychosocial stressors and GERD is of utmost importance. This was also evidenced in the meta-analysis, where patients with a genetic predisposition to anxiety and depression were also more commonly affected by GERD [20]. Thus, GERD may contribute to the development of anxiety and depression, while individuals with these mental health conditions may be more prone to GERD. This increase is probably due to the exhausting fast pace of life, leading to chronic stress, promoting anxiety and depression, harming the mental health of the younger generation, and possibly contributing to GERD [21]. The intricate interplay between detrimental factors, such as reflux events, acidity of refluxate, and esophageal hypersensitivity, versus defensive factors like esophageal acid clearance and mucosal integrity, determines the manifestation and severity of this chronic disorder [22].

Physiologically, stress and anxiety can impact the functioning of the gastrointestinal tract, potentially leading to symptoms associated with GERD. Published literature demonstrates that even short-term stress can influence the composition of the gut microbiome. These bacteria, through their commensal and mutualistic roles, regulate host immune and neurohormonal activity [23]. There is an increasingly recognized role played by gut health in mental health, whereby the gut microbiome communicates with the vagus nerve, immune mediators, and microbial metabolites to influence behavior and attitudes through neurohormonal conduction [24]. On the other hand, the discomfort and disruptions caused by GERD symptoms may contribute to increased stress levels and feelings of depression. This reciprocal relationship highlights the need for a holistic approach when addressing the health of young adults with GERD.

Using similar assessment parameters but considering the cut-off value of 4 on GAD-7 for mild anxiety, hence decreasing the threshold, a study conducted in Beijing Hospital found that 34.9% of GERD patients had anxiety, and 41.3% had depression [25]. In contrast, research carried out in South India showed a much greater incidence of anxiety (66.3%) but a nearly similar incidence of depression (50%) in patients with GERD while using a different scoring system (HAD-S) for evaluating anxiety and depression [13].

GERD patients with coexisting anxiety and depression require a personalized treatment plan, as standardized strategies may not be as effective. According to a study conducted by Nakada et al., the efficacy of proton pump inhibitors (PPIs) in alleviating symptoms of GERD was diminished in individuals with anxiety and depression [26]. A recent best practice update from the American Gastroenterology Association calls on gastroenterologists to adopt a multidisciplinary approach in treating their patients. Having regular assessments and cognitive behavioral therapy administered by qualified mental health providers, and possible inclusion of neuromodulators, such as selective serotonin reuptake inhibitors, may be essential to manage anxiety and depression, alongside standard-of-care treatment for GERD [27]. Hence, it is crucial to identify the anxiety/depression status in patients experiencing GERD symptoms, ensuring proper management, including treatment for both the GERD and psychological status, for optimized therapeutic results.

Our study had a limited sample size of patients from a single center in Pakistan; therefore, the generalizability of our findings may be limited. Additionally, we used consecutive non-random sampling due to financial constraints, which may introduce selection bias. Further exploration into various factors that contribute to GERD, such as lifestyle (dietary habits, stress, occupation, alcohol consumption, and drug use), may provide a deeper understanding of the factors that are closely related to GERD. This study explored a limited number of baseline characteristics and can benefit from a wider assessment of patient profiles. The cross-sectional nature of this study limits the ability to derive causal relationships between GERD and psychosocial factors, which can be explored in future longitudinal studies. The important association of family history and alcohol abuse, which can impact GERD manifestation and its severity, was also not assessed in the present study. While the questionnaires used in this study are standardized and widely accepted in their usability to assess GERD, anxiety, and depression, the outcomes are self-reported. While this is understandable for psychiatric assessments of anxiety and depression, a more quantifiable assessment of GERD could be employed to determine the severity and duration of symptoms and to compare them among the population groups.

## Conclusions

In conclusion, GERD in both young and adult individuals is influenced by various lifestyle factors. The link between GERD and mental health conditions, particularly depression and anxiety, is a complex and bidirectional relationship. Understanding these connections is crucial for developing comprehensive approaches to manage and treat both GERD and associated mental health issues in the younger population.

## Appendices

Appendix 1

**Table 5:** Patient questionnaire form. FSSG: frequency scale for the symptoms of GERD; GERD: gastroesophageal reflux disease; GAD-7: General Anxiety Disorder-7; PHQ-9: Patient Health Questionnaire-9

Variables	Values
Hospital ID	
Age (years)	
Gender	
Education	Up to primary/matric or intermediate/graduate or post-graduate
Socioeconomic status	Lower class/middle class/upper class
BMI	Below 18.5 kg/m^2^/1 8.5-24.9 kg/m^2^/25 or above kg/m^2^
Smoking	Yes/no
FSSG score	-
GERD	Yes/no
If FSSG score is more than 8, then	
GAD-7 score	Anxiety: yes/no
PHQ-9 score	Depression: yes/no

**Table 6:** General Anxiety Disorder-7 (GAD-7) questionnaire.

Over the last two weeks, how often have you been bothered by the following problems?	Not at all	Several days	More than half the days	Nearly every day
1. Feeling nervous, anxious, or on edge	0	1	2	3
2. Not being able to stop or control worrying	0	1	2	3
3. Worrying too much about different things	0	1	2	3
4. Trouble relaxing	0	1	2	3
5. Being so restless that it is hard to sit still	0	1	2	3
6. Becoming easily annoyed or irritable	0	1	2	3
7. Feeling afraid, as if something awful might happen	0	1	2	3
Total points				

**Table 7:** Patient Health Questionnaire-9 (PHQ-9) questionnaire.

Over the last two weeks, how often have you been bothered by the following problems?	Not at all	Several days	More than half the days	Nearly every day
Little interest or pleasure in doing things	0	1	2	3
Feeling down, depressed, or hopeless	0	1	2	3
Trouble falling or staying asleep, or sleeping too much	0	1	2	3
Feeling tired or having little energy	0	1	2	3
Poor appetite or overeating	0	1	2	3
Feeling bad about yourself or that you are a failure or have let yourself or your family down	0	1	2	3
Trouble concentrating on things, such as reading the newspaper or watching television	0	1	2	3
Moving or speaking so slowly that other people could have noticed. Or the opposite, being so fidgety or restless that you have been moving around a lot more than usual	0	1	2	3
Thoughts that you would be better off dead or of hurting yourself	0	1	2	3
Total points				

**Table 8:** Frequency scale for the symptoms of GERD (FSSG) questionnaire. GERD: gastroesophageal reflux disease

S. no.	Questions	Never	Occasionally	Sometimes	Often	Always
1	Do you get heartburn?	0	1	2	3	4
2	Does your stomach get bloated?	0	1	2	3	4
3	Does your stomach ever feel heavy after meals?	0	1	2	3	4
4	Do you sometimes subconsciously rub your chest with your hand?	0	1	2	3	4
5	Do you ever feel sick after meals?	0	1	2	3	4
6	Do you get heartburn after meals?	0	1	2	3	4
7	Do you have an unusual (e.g., burning) sensation in your throat?	0	1	2	3	4
8	Do you feel full while eating meals?	0	1	2	3	4
9	Do some things get stuck when you swallow?	0	1	2	3	4
10	Do you get bitter liquid (acid) coming up into your throat?	0	1	2	3	4
11	Do you burp a lot?	0	1	2	3	4
12	Do you get heartburn if you bend over?	0	1	2	3	4
13	Do you get heartburn?	0	1	2	3	4
14	Total points					

Appendix 2

**Table 9:** Association between BMI and GERD development. ^a^Computed only for a 2x2 table. ^b^0 cells (0.0%) have expected count less than 5. The minimum expected count is 5.13. GERD: gastroesophageal reflux disease

Chi-square tests	Value	df	Asymptotic significance (2-sided)
Pearson chi-square	1.148^b^	1	0.284
Continuity correction^a^	0.616	1	0.433
Number of valid cases	210	-	-

**Table 10:** Association between gender and GERD development. ^a^Computed only for a 2x2 table. ^b^0 cells (0.0%) have expected count less than 5. The minimum expected count is 32.27. GERD: gastroesophageal reflux disease

Chi-square tests	Value	df	Asymptotic significance (2-sided)
Pearson chi-square	2.769^b^	1	0.096
Continuity correction^a^	2.307	1	0.129
Number of valid cases	210	-	-

**Table 11:** Association between smoking status and GERD development. ^a^Computed only for a 2x2 table. ^b^0 cells (0%) have expected count less than 5. The minimum expected count is 11.37. GERD: gastroesophageal reflux disease

Chi-square tests	Value	df	Asymptotic significance (2-sided)
Pearson chi-square	0.022^b^	1	0.882
Continuity correction^a^	0	1	1.000
Number of valid cases	210	-	-

**Table 12:** Association between level of educational attainment and GERD development. ^a^0 cells (0%) have expected count less than 5. The minimum expected count is 9.53. GERD: gastroesophageal reflux disease

Chi-square tests	Value	df	Asymptotic significance (2-sided)
Pearson chi-square	3.886^a^	2	0.143
Likelihood ratio	4.238	2	0.120
Number of valid cases	210	-	-

**Table 13:** Association between socioeconomic class and GERD development. ^a^Computed only for a 2x2 table. ^b^0 cells (0%) have expected count less than 5. The minimum expected count is 16.50. GERD: gastroesophageal reflux disease

Chi-square tests	Value	df	Asymptotic significance (2-sided)
Pearson chi-square	0.274^b^	1	0.601
Continuity correction^a^	0.122	1	0.727
Number of valid cases	210	-	-

Appendix 3

**Table 14:** Odds ratio for anxiety and its association with GERD. ^a^Variable(s) entered on step 1: anxiety on GAD-7 scale. GERD: gastroesophageal reflux disease; GAD-7: General Anxiety Disorder-7

Variable		B	SE	Wald	df	Significance	Exp (B)	95% CI for EXP (B)	
								Lower	Upper
Step 1^a^	Anxiety on GAD-7 scale (1)	21.003	6436.026	0	1	0.997	1323314498.931	0	0
	Constant	0.199	0.154	1.684	1	0.194	1.221	-	-

Appendix 4

**Table 15:** Odds ratio for depression and its association with GERD. ^a^Variable(s) entered on step 1: depression on PHQ-9 scale. GERD: gastroesophageal reflux disease; PHQ-9: Patient Health Questionnaire-9

Variable		B	SE	Wald	df	Significance	Exp (B)	95% CI for EXP (B)	
								Lower	Upper
Step 1^a^	Depression on PHQ-9 scale (1)	5.360	1.026	27.315	1	0	212.800	28.507	1588.533
	Constant	-0.775	0.204	14.408	1	0	0.461	-	-

Appendix 5

**Table 16:** Correlation between anxiety (GAD-7 score) and GERD severity (FSSG score). ^a^Not assuming the null hypothesis. ^b^Using the asymptotic standard error assuming the null hypothesis. ^c^Based on normal approximation. GAD-7: General Anxiety Disorder-7; GERD: gastroesophageal reflux disease; FSSG: frequency scale for the symptoms of GERD

Variables		Value	Asymptotic SE^a^	Approximate T^b^	Approximate significance
Ordinal by ordinal	Kendall's tau-b	0.370	0.046	8.003	0
	Spearman correlation	0.492	0.059	8.156	0^c^
Interval by interval	Pearson's R	0.418	0.062	6.629	0^c^
Number of valid cases		210	-	-	-

**Table 17:** Correlation between depression (PHQ-9 score) and GERD severity (FSSG score). ^a^Not assuming the null hypothesis. ^b^Using the asymptotic standard error assuming the null hypothesis. ^c^Based on normal approximation. PHQ-9: Patient Health Questionnaire-9; GERD: gastroesophageal reflux disease; FSSG: frequency scale for the symptoms of GERD

Variables		Value	Asymptotic SE^a^	Approximate T^b^	Approximate significance
Ordinal by ordinal	Kendall's tau-b	0.327	0.047	6.948	0
	Spearman correlation	0.442	0.061	7.106	0^c^
Interval by interval	Pearson's R	0.415	0.070	6.582	0^c^
Number of valid cases		210	-	-	-

Appendix 6

**Table 18:** Observed and expected counts of patients with anxiety and educational attainment. GAD-7: General Anxiety Disorder-7

Variables			Anxiety on GAD-7 scale	
			No	Yes
Education	Upto primary	Count	14	12
		Expected count	21.2	4.8
	Matric or intermediate	Count	86	14
		Expected count	81.4	18.6
	Graduate or post-graduate	Count	71	13
		Expected count	68.4	15.6

**Table 19:** Chi-square test showing association between anxiety and educational attainment. ^a^Cells (16.7%) have expected count less than 5. The minimum expected count is 4.83.

Variables	Value	df	Asymptotic significance (2-sided)	Exact significance (2-sided)
Pearson chi-square	14.994^a^	2	0.001	0.001
Fisher-Freeman-Halton exact test	12.472	-	-	0.002
Number of valid cases	210	-	-	-

Appendix 7

**Table 20:** Multivariate logistic regression for GERD and relation to anxiety. ^a^Variable(s) entered on step 1: anxiety on GAD-7 scale, gender, smoking, BMI, socioeconomic status, and education. GERD: gastroesophageal reflux disease; GAD-7: General Anxiety Disorder-7

Variables		B	SE	Wald	df	Significance	Exp (B)	95% CI for EXP (B)	
								Lower	Upper
Step 1^a^	Anxiety on GAD-7 scale (1)	21.210	6275.830	0	1	0.997	1626884348.560	0	0
	Gender (1)	-0.773	0.365	4.475	1	0.034	0.462	0.225	0.945
	Smoking (1)	0.833	0.497	2.809	1	0.094	2.300	0.868	6.090
	BMI (1)	1.684	0.845	3.973	1	0.046	5.386	1.029	28.200
	Socioeconomic status (1)	-0.303	0.404	0.564	1	0.453	0.738	0.335	1.629
	Education	-	-	0.477	2	0.788	-	-	-
	Education (1)	-0.207	0.620	0.111	1	0.739	0.813	0.241	2.742
	Education (2)	-0.374	0.624	0.359	1	0.549	0.688	0.202	2.338
	Constant	-0.701	1.029	0.465	1	0.495	0.496	-	-

**Table 21:** Multivariate logistic regression for GERD and relation to depression. ^a^Variable(s) entered on step 1: depression on PHQ-9 scale, gender, smoking, BMI, socioeconomic status, and education. GERD: gastroesophageal reflux disease; PHQ-9: Patient Health Questionnaire-9

Variables		B	SE	Wald	df	Significance	Exp (B)	95% CI for EXP (B)	
								Lower	Upper
Step 1^a^	Depression on PHQ-9 scale (1)	5.420	1.035	27.431	1	0	225.769	29.708	1715.781
	Gender (1)	-0.474	0.489	0.939	1	0.333	0.623	0.239	1.623
	Smoking (1)	0.964	0.623	2.392	1	0.122	2.621	0.773	8.888
	BMI (1)	1.043	1.021	1.044	1	0.307	2.839	0.384	21.004
	Socioeconomic status (1)	-0.019	0.539	0.001	1	0.971	0.981	0.341	2.821
	Education	-	-	2.002	2	0.368	-	-	-
	Education (1)	-1.032	0.736	1.967	1	0.161	0.356	0.084	1.507
	Education (2)	-0.741	0.721	1.056	1	0.304	0.477	0.116	1.959
	Constant	-0.899	1.210	0.552	1	0.457	0.407	-	-
